# Hemoglobin-based oxygen carrier: what we have learned and where we are heading?

**DOI:** 10.1093/procel/pwag019

**Published:** 2026-03-24

**Authors:** Han Xiao, Weiliang Xia

**Affiliations:** School of Life Sciences and Biotechnology, Shanghai Jiao Tong University, Shanghai 200240, China; Antai College of Economics and Management, Shanghai Jiao Tong University, Shanghai 200030, China; Center for Aging and Cancer Research, Global Institute of Future Technology, Shanghai Jiao Tong University, Shanghai 200240, China; School of Biomedical Engineering, Shanghai Jiao Tong University, Shanghai 200240, China; State Key Laboratory of Systems Medicine for Cancer, Renji Hospital, School of Medicine, Shanghai Jiao Tong University, Shanghai 200127, China

## The urgent need of red blood cells

Hundreds of millions of patients are suffering from traumatic injury and obstetric bleeding worldwide (Liu et al., 2022; Raykar et al., 2021). Among those who do not reach the hospital for blood transfusion, hemorrhage turns out to be the primary cause of mortality, yet still accounts for up to 40% of deaths of those who do, with the exact number of 1.9 million per year worldwide (Chambers et al., 2019; Jahr, 2022; Raykar et al., 2021). Red blood cells (RBCs) are the most frequently transfused blood component. Their depletion poses a severe mortality risk, as every 10 g/L decrease from a 50 g/L baseline in their core component, hemoglobin, doubles the probability of adverse effects such as respiratory failure and myocardial infarction (García-Roa et al., 2017; Guinn et al., 2018; LaPar et al., 2018; Liu et al., 2022; Pattakos et al., 2012). Despite the severe consequences, timely RBC transfusion has been a struggle for decades. To date, the best approach to on-site hemorrhage management has been limited to isotonic crystalloid solutions, which do not deliver oxygen into the bloodstream. Even worse, with the rising tension of ongoing wars and the revelation of the contaminated blood scandal, our need for safe on-site RBC transfusion has never been greater. With all elements considered, causes of this huge unmet medical need can be concluded as the following: lack of supply, logistical barriers, and factors entailing application issues such as rare blood types and transfusion refusal.

As for the lack of supply, nearly two-thirds of countries worldwide are in dearth of sufficient blood to meet the clinical demand, especially in low- and middle-income countries (LMICs) where blood banks depend on paid or replacement donations (Jacobs Jeremy et al., 2024; Raykar et al., 2021). Even the United States, the largest blood supplier country, also suffers from seasonal shortage and potential HIV contamination (García-Roa et al., 2017; Hébert Paul et al., 1999; Liu et al., 2022). While China, the second most populous country, exhibits a blood donation rate of mere 11.4‰, and sometimes depends on mutual donation to safeguard emergency blood needs (NHCOTPSRO China, 2025; Xue et al., 2016). As for logistical barriers, issues entailing storage requirements and storage lesion remain unsolved. While the short shelf life of 35–42 days impedes the static storage to offset occasional shortage, strict requirements for cold storage make portable transport to the site of delivery, such as an ambulance, a challenge (DARPA, 2025; DBT Office, 2021; Sen Gupta, 2019). Storage lesion, on the other hand, can cause metabolic decay and membrane deformity that impair perfusion and oxygenation efficacy (Banka et al., 2019; García-Roa et al., 2017; Goel et al., 2016; Remy et al., 2015). Application issues also impede the on-site delivery of fresh RBCs. Multiple blood types and pre-transfusion matching require more portable reserves and more complex pre-transfusion preparation. Patients with rare blood type, sickle cell anemia and religious refusal further complicate the application process.

## Ways to get more “red blood cells”

Based on the hindrance aforementioned, a portable, universal, easy-to-preserve alternative of human red blood cells was proposed to solve these problems, with three main techniques making progress: perfluorocarbons, manufactured RBCs, and hemoglobin-based oxygen carrier (Table 1).

**Table 1.** Summary of current solutions for *in vivo* oxygen delivery.

Oxygen carriers	Origin	Design methods	Advantages	Disadvantages	Future development goals
**Natural RBCs**	Donated blood	Extraction, purification, and classification	Standard of care Biocompatible	Short shelf life Strict storage requirements, hardly portable Rare blood type issue Occasional shortage	Novel storage methods Higher donation rate Robust blood bank network
**PFC (perfluorocarbon)**	Chemical compound	Stabilization in emulsion	Universal application and portability Better oxygen dissolution concentration Properties and functions resistant to physical parameters Available for mass production	Oxygen release before reaching target issue Supplemental oxygen inhalation needed Short stability time Low clearance rate and co-factor deposition Considered a potent pollutant in mass production Interference with pulse oximetry equipment and chemistry analytes Ethical concern	Smaller and more stable emulsions Biocompatible surfactant Nanomaterial encapsulation
**Manufactured RBCs**	Patient-derived blood	HSPCs differentiation or an immortalized erythroid progenitor cell line	Biocompatible Closest to natural RBCs Available for gene editing	Short shelf life Strict storage requirements, hardly portable Restricted capacity for expansion and unsatisfying enucleation rate Industrial mass production bottleneck and high cost	Novel storage methods Higher industrial productivity
**HBOC (hemoglobin-based oxygen carrier)**	Obsolete animal or human blood	Multiple chemical modifications to hemoglobin	Universal application and portability Available for modification to adjust product attributes Solid principle based on natural hemoglobin Available for mass production	Potential immunization issue and reperfusion injury Affected by pharmacological and environmental factors Short half-life Significant adverse effects if unsterilized Ethical concern	Novel modification methods Further safety assessment Appropriate indication for use

The first approach is perfluorocarbons (PFCs), which are chemical compounds designed to dissolve oxygen as a gas carrier. It can be applied as a universal solution and stored on a large scale (Khan et al., 2020). However, early PFC emulsion products such as Fluosol-DA and Fluosol-43 had been withdrawn by the FDA either due to inadequate oxygen uptake, long organ retention, or difficulty of frozen storage and thawing prior to use (Castro and Briceno, 2010; Thyes and Spahn, 2005). Subsequent products such as Oxygent and HemaGen with a more stable emulsifier and optimized PFC content were suspended due to severe adverse effects such as higher incidence of stroke and adverse neurological outcomes (Castro and Briceno, 2010; Zhao et al., 2024).

The second approach is to manufacture RBCs through iPSC technology. It was proposed to maneuver the differentiation and expansion capacity of upstream RBC progenitors, such as the immortalized adult erythroid progenitor cell line (Bristol Erythroid Line Adult, BEL-A), in order to achieve the mass production of RBCs *ex vivo* (Kupzig et al., 2017; Trakarnsanga et al., 2017). However, it is still in its infancy under early clinical trials, with limited capacity and high costs due to unsatisfying enucleation rates and bioreactor yield bottlenecks (Heshusius et al., 2019; Kweon et al., 2023; Lee et al., 2023; Pellegrin et al., 2021). Furthermore, positioned as an industrial way to manufacture RBCs, the storage problem and erythrocyte alloimmunization issues of the obtained RBCs remain intractable.

The third approach, hemoglobin-based oxygen carriers (HBOC), is a general product that modifies and utilizes hemoglobin in biocompatible ways on the basis of its oxygen-binding properties. Hemoglobin can be obtained from outdated human blood or animal origin, making it available for a sustainable supply. Biochemical modifications can enhance its stability and portability, while the elimination of blood group antigens ensures its universality.

Despite decades of pursuit, no HBOC has been commercialized as a safe blood substitute. However, lessons learned from past failures have illustrated that the bottleneck for HBOC’s approval no longer lies in the technology itself. With a promising clinical profile, the latest HBOC is poised for commercialization to address the aforementioned RBC issues. In this commentary, we introduce the basic mechanisms of different HBOCs and go through the development of three generations of HBOCs. As past failures have shown that gaining approval of HBOC is not a simple matter of developing a viable product, we conclude these precious lessons, highlight areas of consensus and outline important aspects of HBOC in clinical approval. We also specify the implication for use of HBOC in real-world settings to bridge the gap from clinical endpoints to commercial use scenario, and propose further development with regard to its overlooked properties for future investigators, regulators, and potential investors.

## Hemoglobin: the key component of HBOC

Hemoglobin (Hb) is the key component, both in HBOC and natural RBC, that fulfills the primary function of oxygen delivery as a gas carrier. It is a 64-kDa tetramer of two α- and two β-polypeptide chains, where central iron atoms enable reversible oxygen binding. The dynamic affinity with oxygen of Hb is mediated through the microenvironment and 2,3-diphosphoglycerate(2,3-DPG), an allosteric effector molecule inside RBC (Khan et al., 2020; Sen Gupta, 2019). 2,3-DPG binds to a specific allosteric site between the two β-subunits of deoxyhemoglobin, stabilizing the low-affinity T (tense) state. This increases the energy barrier for transitioning to the high-affinity R (relaxed) state, and effectively raises the oxygen concentration required for Hb to become saturated. The rise of 2,3-DPG concentration right-shifts the oxygen-hemoglobin dissociation curve and facilitates the oxygen release at higher O_2_ partial pressure (Fig. 1).

**Figure 1.**
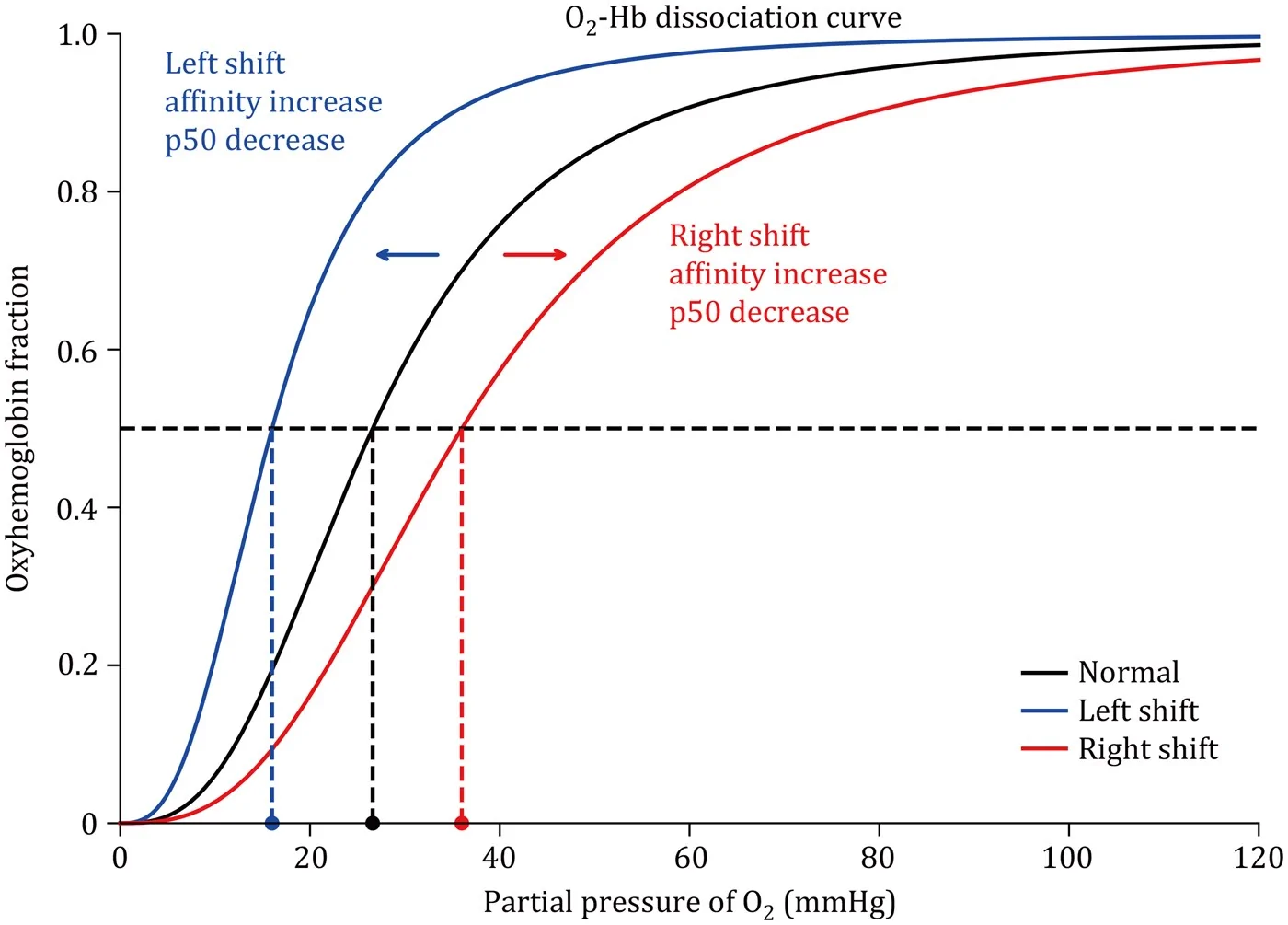
**The oxygen-hemoglobin dissociation curve**. P_50_ denotes the partial pressure of oxygen at which hemoglobin becomes 50% saturated with oxygen. It is inversely related to the oxygen-binding affinity of hemoglobin, which is normally influenced by pH, CO_2_ tension, and enzymes such as 2,3-DPG. Standard P_50_ in humans is 26.9 mmHg at pH 7.4 and 37°C (Balcerek et al., 2020). A lower P_50_ denotes a higher affinity, whereas a higher P_50_ indicates a lower affinity and increased oxygen release to tissues.

Microenvironment also plays a pivotal role in the regulation of Hb (Bellingham et al., 1971). In systemic capillaries where CO_2_ pressure is high and pH is relatively low, the intake of CO_2_ and high concentration of H^+^ facilitate the binding between hemoglobin and protons, leading to the propensity of oxygen releasing and a higher partial pressure of O_2_(P_50_) and *vice versa* (Fig. 2). The concept of the dynamic affinity to facilitate the function of carrier has been employed in some of the latest HBOC products.

**Figure 2.**
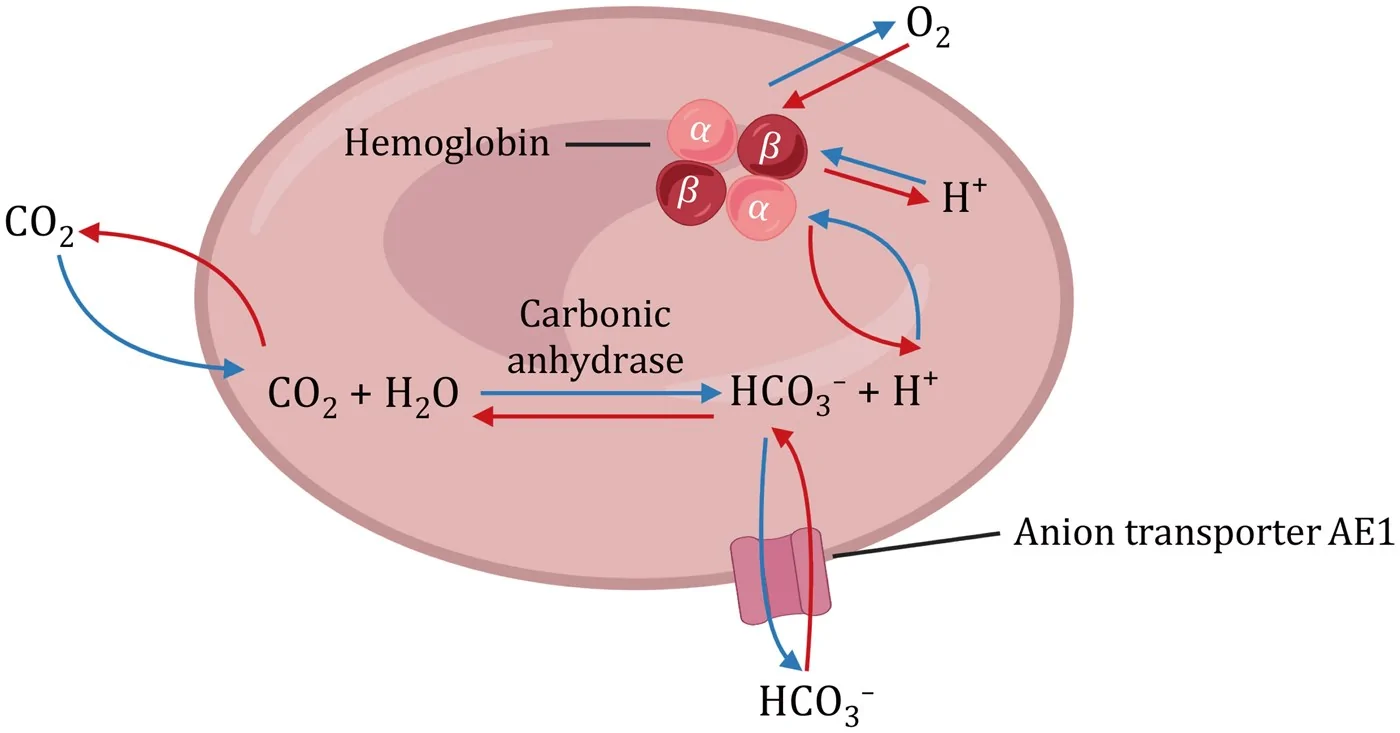
**The regulation of oxygen affinity through microenvironment**. Elevated concentrations of H^+^ ions prompt the binding of protons with hemoglobin, resulting in a shift from the R to the T conformation. This process is further facilitated by carbonic anhydrase, which converts CO_2_ into bicarbonate and protons via carbonic acid. (Blue arrows represent systemic capillary environment where pH is relatively low; red arrows represent pulmonary capillary environment where oxygen pressure is high.) Created with Biorender.com.

## Learning from hemoglobin: early attempts for the first generation of HBOC

Since hemoglobin does not possess blood group antigens and lacks a membrane, early attempts were made to purify Hb as a simplified universal oxygen carrier (Sen Gupta, 2019; Tsai et al., 2004). Normally, the preliminary processed Hb solution is referred to as the first-generation HBOC (Burhop, 2022). However, without a protective membrane, Hb is prone to disintegrate into dimeric or monomeric subunits, leading to a much shorter circulation due to rapid extravasation into the extravascular space and subsequent renal clearance (Buehler et al., 2010; Schechter and Gladwin, 2003). Tiny subunits that slip through the vascular crevice may also cause endothelial scavenging of nitric oxide (NO), which leads to vasoconstriction (Amberson et al., 1934; Buehler et al., 2010; Kim-Shapiro et al., 2006; Schechter and Gladwin, 2003). Besides, cell-free Hb is devoid of regulatory enzymes (e.g., 2,3-DPG) and protective antioxidants, leaving it vulnerable to undesired radicals and MetHb formation (Cherian, 2022; Khan et al., 2020; Sen Gupta, 2019; Umbreit, 2007).

Developing a viable HBOC is not a simple matter of creating an oxygen-binding tool. Experimental findings prompted scientists to reanalyze the role of auxiliary molecules within natural RBCs and the complex microenvironment HBOC may interact with. In hindsight, the early attempts for the first-generation HBOC can be deemed as explorations towards the unknown, whose failure was inevitable with the lack of basic comprehension of the biochemical properties of natural RBC and its physiological interactions within the microvasculature, yet the rudimentary knowledge of metabolism and physiology obtained paved the way for future HBOC development.

## The second generation of HBOC: mature techniques with over-ambitious goals

Given the promising attributes of oxygen binding yet highly-reactive and toxic nature of cell-free Hb, corresponding techniques have been employed to optimize the first generation Hb solution. The second generation HBOCs are semisynthetic complexes based on natural Hb while utilizing chemical or other modification methods to prevent the cell-free issues aforementioned from happening. Widely used modifications can be classified as cross-linking, polymerization, conjugation, and encapsulation (Table 2).

**Table 2.** Methods of modification for HBOCs. Created with Biorender.com.

Modification	Legend	Method	Representative products
**Crosslinking**	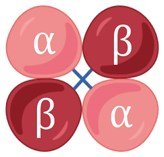	Crosslinking can be performed in both an intermolecular or intramolecular way. Normally, we refer to the internal linkage as crosslinking specifically. The α-α and β-β chains of diaspirin or raffinose stabilize the structure and prevent the formation of αβ dimers or monomeric subunits. It focuses on the internal reinforcement of rigidity and has been most widely applied to the synthesis.	PolyHeme (Northfield Laboratories) HemAssist (Baxter Corporation) HemoLink (Hemosol Inc.) Optro (Somatogen Inc.) YQ23 (Bios Biotechnology) …
**Polymerization**	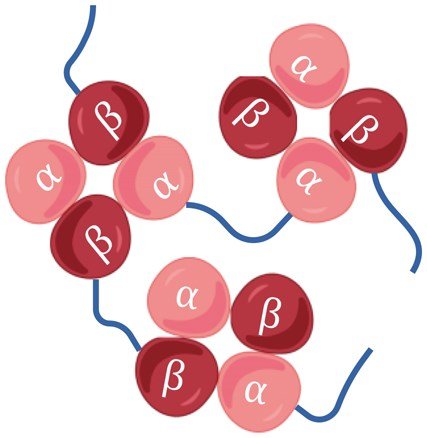	Polymerization focuses on the intermolecular binding. Surface amino acid groups are usually linked by reagents like glutaraldehyde or O-raffinose. The external linkage bulk up the size, thereby mitigating the risk of penetrating through the endothelial layer.	Hemopure (HbO2 Therapeutics LLC.) PolyHeme (Northfield Laboratories) OxyVita (OXYVITA Inc.) …
**Conjugation**	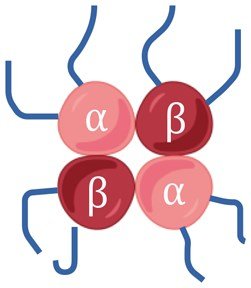	Conjugation applies large molecules, such as antioxidant enzymes or other allosteric effectors, to the surface groups of hemoglobin. Conjugated molecules increase the size and therefore ensure its intravascular stay. Common enzymes chosen to be conjugated are catalase (CAT) and superoxide dismutase (SOD), which may protect the molecules from oxidative stress and free radicals.	Hemospan (Sangart Inc.) HemoxiMer (Apex Bioscience) …
**Encapsulation**	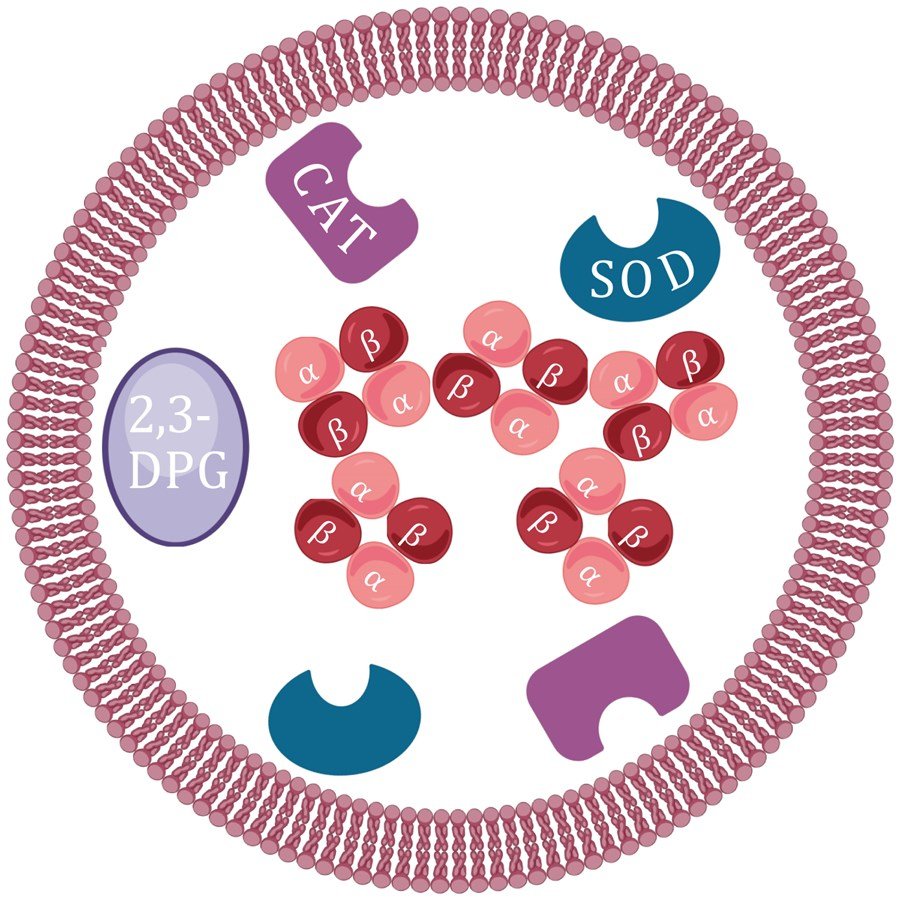	Encapsulation makes use of biochemical or synthetic compounds to package natural Hb. By shielding the hemoglobin, the particle can be protected from plasma-induced effects, which mimic the function of the natural membrane. Essential enzymes such as 2,3-DPG, MetHb reductase, CAT, and SOD can be co-encapsulated in order to imitate the physiological state of natural RBCs. Normally, it shows similar oxygen equilibrium curves to RBCs. Common compounds chosen for the outer layer are phospholipids and cholesterol.	HbV (Dr. Sakai; Nara Medical University) ErythroMer (KaloCyte Inc.) …

For these modifications, the second-generation HBOC has made great strides, with several products flourishing into clinical phase III investigation, such as Hemopure, PolyHeme, HemeAssist, Hemospan, etc. (Liu et al., 2022; Mohanto et al., 2025). Sadly, all products of the second generation fell at the final hurdle, among which, Hemopure and PolyHeme were the most investigated, whose failure could be seen as the epitome of the second generation HBOCs, providing profound food for thought (Table 3).

**Table 3.** Pipelines of some of the most representative HBOCs and their current statuses.

	Product	Current status	Clinical indication	Timeline	References/registration number
**Second generation HBOC**	HBOC-201 (Hemopure)	BLA denied Available for compassionate use 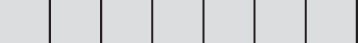	Life-threatening anemia (blood substitute)	Expanded Access Study completed in 2023	NCT01881503
	PolyHeme	BLA denied 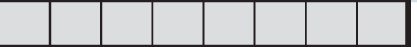	Acute blood loss from trauma (superiority and non-inferiority assessment)	Denied in 2009	Moore et al. (2009)
	HemoLink	Phase III terminated 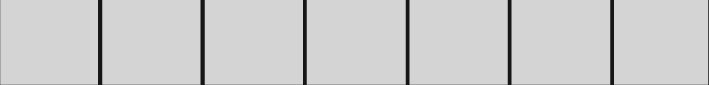	Acute normovolemic hemodilution (blood substitute)	Terminated in 2004	NCT00038454
	HemAssist	Phase III terminated 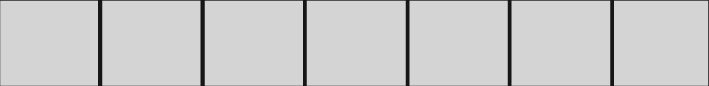	Hemorrhagic shock	Terminated in 1999	Kerner et al. (2003)
	PHP (Hemoximer)	BLA denied 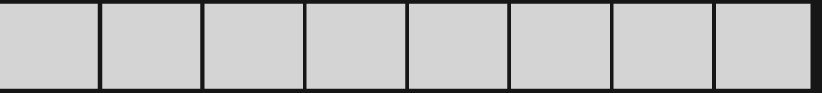	NO-induced distributive shock	Denied in 2014	NCT00021502
	MP4 (Hemospan)	BLA denied 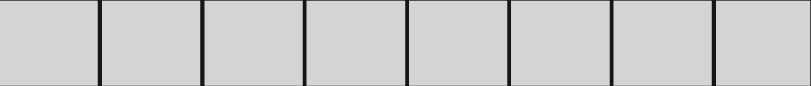	Oxygen therapeutic (increase oxygen delivery to at-risk tissues)	Development shelved in 2015	NCT00421200 NCT00420277
**Third generation HBOC**	PP-007 (Sanguinate)	Phase II recruiting (fast track) 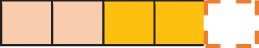	Acute ischemic stroke	Phase I results presented in Feb-2025	NCT04677777
	NMU-HbV	Phase Ib recruiting 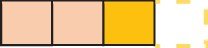	Life-threatening massive hemorrhage	Phase Ib initiated in Jan-2025	jRCT2051240249
	OxyVita	Pre-clinical investigation 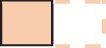	Improve oxygen delivery in hemodilution	Latest investigational article was published in 2017	Wollocko et al. (2017)
	ErythroMer	Pre-clinical investigation 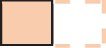	Hemorrhage	IND scheduled before 2027 (DARPA granted in 2023 with a four-year plan)	DARPA-FSHARP program
	PNPH (SanFlow)	Pre-clinical investigation 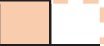	Multifunctional therapeutic gas carrier	Latest investigational article was published in 2023	Wang et al. (2023)
	YQ23	Phase Ib/IIa terminated 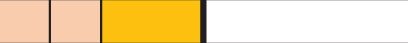	Hemorrhage	Terminated in 2023 due to a business decision	NCT04792008
	XY101	Appling IND 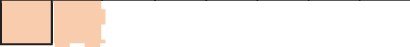	–	Start applying for IND from 2024	CXSL2400101
	HEMO_2_life	Approved by the European Union 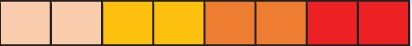	Organ preservation	Approved in 2022	NCT04181710

Clinical progress is indicated by different colors with the number of squares (eight in total), with different shades of red representing preclinical, stage I, II, and III, respectively. Each color contains two small squares, i.e., different stages of the same clinical phase, e.g., phase a, b. Gray indicates clinical failure (the last square of Hemopure is specifically marked as compassionate use despite disapproval), the thick black line indicates termination of progress, and the dotted line indicates moving on to the next phase.

### Hemopure

Hemopure from HbO2 Therapeutics LLC. is a cell-free, glutaraldehyde cross-linked and polymerized bovine Hb in a modified lactated Ringer’s solution (Table 4). Like any other second-generation HBOC, Hemopure was positioned with an over-ambitious goal: to serve as an alternative to natural RBCs (Jahr et al., 2008; Waters et al., 2022). The excess confidence and irrational focus on replacing natural RBCs prompted a high-risk phase III clinical design. It randomized orthopedic surgery patients to receive Hemopure (up to 10 units) or packed RBCs, aiming to demonstrate safety, non-inferiority, and the elimination of RBC transfusion needs in at least 35% of patients. Natural RBCs were reserved solely as a rescue therapy when Hemopure tolerance or efficacy limits were exceeded.

**Table 4.** Characteristics of human RBCs and its contrast with representative HBOCs from publicly available peer-reviewed articles.

	Product	Modification	Origin	Average weight (kDa)	Particle size (nm)	pH	Hb concentration (g/dL)	P_50_ (mmHg)	COP (mmHg)	Half life	Shelf life	References
**Second generation HBOC**	Natural RBCs	–	Bovine	64	7,000	7.4	11–16	27	2	25–40 days	4°C: <5 weeks	
	HBOC-201 (Hemopure)	Glutaraldehyde cross-linked and polymerized	Bovine	250	8	7.6–7.9	12–14	40	25	19 h	4–21°C: 3 years	Jahr et al. (2022), Waters et al. (2022)
	PolyHeme	Glutaraldehyde polymerized	Human (outdated)	150	–	–	10	26–32	20–25	24 h	4–8°C: >1 year 21°C: 6 weeks	Jahr et al. (2022)
	HemoLink	o-raffnose-polymerized, cross-linked Hb	Human (outdated)	64–500	6.4–13	7–8	10	39 ± 12	25.4	18–20 h	4°C: >1 year	Jahr et al. (2022), Mohanto et al. (2023), Carmichael et al. (2000)
	HemAssist	Diasprin a-a cross-linked Hb	Human (outdated)	64	6.4	7.4	10	32	42	2.5–3.3 h	<5°C: 1+ years	Jahr et al. (2022), Przybelski et al. (1996)
	PHP (Hemoximer)	Pyridoxalated Hb Polyoxyethylene conjugate	Human (outdated)	109	–	7.4	8	23.4	49	–	–	Jahr et al. (2022), Priavalle and De Angelo (2022)
	MP4 (Hemospan)	PEGylated Hb plus maleimide	Human (outdated)	90	13–19	7.3–7.7	4.2	6	55	42.8–66.2 h	–	Matsuhira and Sakai (2022), Björkholm et al. (2005)
**Third generation HBOC**	PP-007 (Sanguinate)	PEGylated carboxy-Hb	Bovine	109–120	–	8	4–5	7–16	–	7.9–13.8 h	48°C: 4 years 21°C: 2 years	Misra et al. (2014), Jahr et al. (2022)
	NMU-HbV	Encapsulated in a PEGylated phospholipid vesicle	Human (outdated)	–	250–280	–	10	15	≈0	15–20 h	21°C: >2 years	Sakai et al. (2022), Matsuhira and Sakai (2022)
	OxyVita	Zero-linked polymerization	Bovine	17,000–33,000	72	7.5	6	4–6.4	3	72 h	Lyophilized: 5 years Liquid (21°C): 1 year	Wollocko et al. (2022), Wollocko et al. (2020)
	ErythroMer	Encapsulated in a peptide-lipid amphiphile-based nanoparticle	Human (outdated)	–	100–140	7.4	–	pH = 7.2: 30 pH = 7.4: 22 pH = 7.6: 19	–	–	25°C: 3 months 4°C: 2 years	Mittal et al. (2022)
	PNPH (SanFlow)	Polynitroxylated PEGylated Hb	Bovine	–	8	7.4	4	10–12	–	–	2 years	Hsia and Ma (2012), Shellington et al. (2011)
	YQ23	Non-polymerized, cross-linked, cell-free, tetrameric Hb	Bovine	64	–	7.3–7.6	5–10	40	–	–	–	Kuang et al. (2021), Li et al. (2017), Zhang et al. (2021), Kuang et al. (2019), Lee et al. (2015)
	HEMO_₂_life	Derived from a large extracellular Hb molecule	Marine invertebrate	3,648 ± 24	30	6.5–7.5	4	7	1	2.5 days	−20°C ± 5°C/−80°C ± 10°C: 5 years	Zal et al. (2022), Matsuhira and Sakai (2022)

The clinical trial was supposed to simulate situations where Hemopure was used until erythrocytes became available to replace moderate blood loss for elective surgery. However, the primary endpoint of reduced transfusion requirement did not directly reflect the value of bridging the gap before RBC became available. Besides, RBCs were transfused immediately in the control group, which reflected the current best solution in the resource rich environment and indirectly led to a head-to-head data analysis between PRBC and Hemopure only. As a result, the head-to-head safety endpoints of Hemopure was poorly met against natural RBC, which came as no surprise (Jahr et al., 2008). The seemingly unintended mismatch between clinical design and use scenario reflected its over-ambitious goal, as the evaluation only oriented at Hemopure alone while RBC infusion included in the treatment group was only designed as the fallback. As the necessary following-up procedure, RBC transfusion should also be included and interpreted with Hemopure as a whole.

### PolyHeme

PolyHeme from Northfield Laboratories is a pyridoxylated, crosslinked Hb from outdated RBCs with glutaraldehyde (Cralley and Moore, 2022). The final phase III trial of PolyHeme opted to compare HBOC with crystalloid as the resuscitation fluid before blood became available. Patients with a systolic blood pressure ≤90 mmHg were randomized to receive field resuscitation with PolyHeme or crystalloid. Allogenic blood was immediately transfused as needed once patients reached the trauma center, but it could only be applied after the six units of maximum use of PolyHeme during the first 12 h (Cralley and Moore, 2022; Moore et al., 2009).

Although PolyHeme was not compared directly to natural RBC, the immature clinical design and its resulting unmet clinical endpoints still led to its disapproval. The average time to necessary blood transfusion was compared to analyze whether PolyHeme could delay the blood transfusion, meanwhile, the threshold of 12 h limitation was deliberately set in the experimental group to mimic the situation where blood is unavailable. Although the average time of PolyHeme group was indeed significantly longer (14.1 h vs. 1.5 h), the efficacy of PolyHeme could still not be assured since RBCs were strictly forbidden during the first 12 h. Instead of reflecting a problematic scenario in military and rural environments as intended, the median time to arrival at the treatment facility for both cohorts was only 26 min, indicating robust trauma care systems and resource abundant environments. Moreover, only half of the control subjects were in need of blood transfusions, reflecting a population in which PolyHeme would not be indicated for (Cralley and Moore, 2022; Moore et al., 2009). The failure to reflect a resource-constrained scenario and the recruitment of patients with low needs made it harder for PolyHeme to showcase its efficacy and true value against the current SOP. As a result, the safety profile of noninferiority was poorly met, which may be partly explained by the defects in the trial design and recruitment rather than the pitfalls in the product itself.

In 2009, the FDA declined the application of PolyHeme and the company filed for bankruptcy. The failure of PolyHeme reflected the thorny dilemma of how to incorporate the inaccessibility to blood in real-world settings without raising any ethical concern. Certain endpoints and trial designs should be carefully mulled over, so as to extrapolate the true value of HBOC in a resource-restrained environment.

The failure of the second-generation HBOC has left several legacies for the successor products. It has been shown that the naturally endowed magic of allogeneic RBCs could hardly be surpassed either from the efficacy or safety prospect. Appropriate marker of efficacy for HBOC may lie in the pre-hospital settings, where HBOCs are compared with crystalloid administration or no transfusion available at all to reflect its capability. The disapproval of the second-generation HBOC is a pity, as it indeed demonstrated promising value to solve the unmet medical needs in certain conditions, but failed due to the overall mismatch in product positioning as well as the immature clinical design that could have been prevented. This explains why the FDA granted compassionate use of Hemopure while the US military was still sponsoring its clinical trials in South Africa (Waters et al., 2022). The defects identified in the initial attempts have informed the subsequent developments of the third generation of HBOC.

## Resorting to nature with clearer views: advances in the third generation of HBOC

Lessons learned from the past two generations have led to a conceptual change that HBOC should be positioned as an addition to the current SOP, rather than as a complete overhaul. Subsequent development has therefore altered the focus on the pre-clinical and en route settings with less ambitious goals. Some products choose to target a niche but pragmatic market before further expanding their implications for use on the bedside. Refined engineering techniques also allow further emulation towards natural RBCs, bringing HbV, ErythroMer, and HEMO_2_life into the spotlight.

### HbV

Encapsulation was one of the first attempts made by investigators to establish an artificial RBC, as it emulates the structure of the natural one while avoiding most of the defects of acellular hemoglobin, aforementioned. However, new issues entailing biocompatibility, storage stability, and cost of scaling with regard to low encapsulation efficiency had impeded the development of encapsulated HBOC until the advent of HbV.

Hemoglobin vesicles (HbV) from Dr. Sakai’s team are a cellular-structured HBOC encapsulating a purified outdated human hemoglobin in a pegylated phospholipid vesicle. Four specific lipids were selected to ensure its biocompatibility and structure flexibility, while polymerized phospholipid theoretically endows HbV a longer storage time and shields the toxicity of Hb (Azuma et al., 2022; Kure and Sakai, 2021; Sakai et al., 2022). Essential enzymes such as catalase are encapsulated, and the P_50_ of HbV is adjusted to a relatively low level of 15 mmHg by co-encapsulating equimolar pyridoxal 5′-phosphate (PLP), an allosteric effector, to HbV (Sakai et al., 2022). As for the cost, a kneading method using an optimized rotation-revolution mixer has been developed to facilitate lipid dispersion and liposome formation, which greatly increases the encapsulation efficiency (Kure and Sakai, 2021). Of note, HbV can utilize Hb’s nature of CO binding and functions as the CO carrier before releasing CO to CO-scavenged vessels triggered by oxygenation (Sakai et al., 2022). Though not proven in clinical settings, it may provide a new therapy for anti-inflammatory and anti-oxidative protection and can be theoretically co-applied to mitigate possible vasoconstriction (Fig. 3).

**Figure 3.**
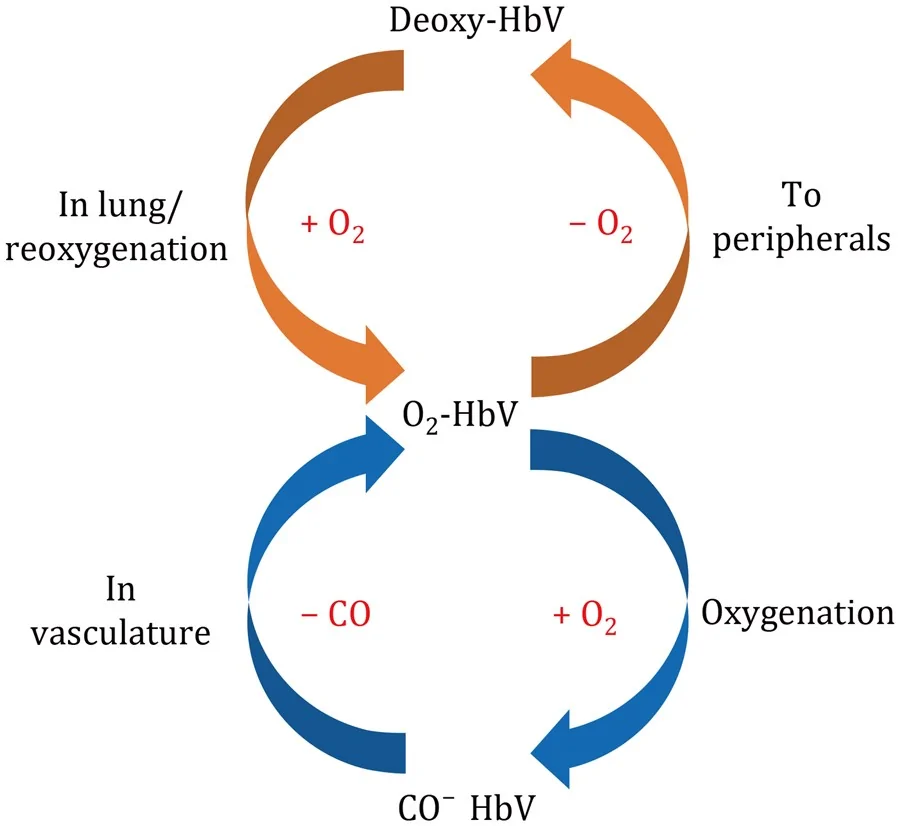
**Pathways for HbV to function as oxygen and carbon monoxide carriers**. CO-HbV is the precursor of HbV and can function as the CO carrier by releasing CO to CO-scavenged vessels triggered by oxygenation. The resultant, O_2_-HbV, acts as the common oxygen carrier and turns to deoxy-HbV during reoxygenation in the lung. After oxygenation before injection or in the lung, it converts into O_2_-HbV and turns back to deoxy-HbV, ready for the next cycle if not metabolized (Sakai et al., 2022). Adapted from (Sakai et al., 2022).

Following solid efficacy in pre-clinical studies, HbV was brought to the bedside in 2020. The phase I clinical trial demonstrated relatively acceptable adverse effects and the ability of HbV to safely function as an oxygen carrier in blood vessels (Azuma et al., 2022). Researchers claim to put HbV into practical use around 2030 and have started to solicit volunteers for the next trial based on the encouraging results obtained from the initial study (Peckitt, 2024).

### ErythroMer

In 2023, the Defense Advanced Research Projects Agency (DARPA) announced a $46 million grant in order to develop a stable, field-deployable blood substitute, with ErythroMer set as its core (DBT Office, 2021; Zaleski, 2024). ErythroMer (EM, KaloCyte Inc.) is a self-assembled, peptidic-lipid amphiphile-based nanoparticle that encapsulates outdated human Hb. Different from the artificial bilayers in liposomal-based HbV, the membrane of ErythroMer consists of the main components of natural RBC shell while incorporating pH-sensitive amines that modulate Hb allosteric effectors (Fig. 4). The hydrophilic “head” of the construct is designed to boost its biocompatibility and *in vivo* degradation, and the hydrophobic fatty acid, on the other end, attenuates NO diffusion (Mittal et al., 2022). The most distinctive characteristic of EM is its pH-determined P_50_, which rises to 30 mmHg under lower pH (7.2) and reduces to 19 mmHg under higher pH (7.6), imitating the accommodating nature of the heterotropic effector system in natural RBCs. Theoretically, it can enhance O_2_ transportation efficiency and mitigate oxygenation damage (Mittal et al., 2022; Zaleski, 2024). For instance, more oxygen can be bound in the lung due to its high pH environment, and more oxygen is prone to offload in the periphery and trauma tissues as needed in the low pH environment. In particular, EM makes use of the pH-responsive nature of the synthetic amine, KC1003, which is incorporated in the membrane, and triggers the replacement of 2,3-DPG that is bound to KC1003 due to amine protonation, resulting in 2,3-DPG ∼ Hb locking up and right-shift the affinity curve. As pH rises, 2,3-DPG re-associates with increasingly available KC1003 due to ionization (Mittal et al., 2022). Besides, EM is available for sterile lyophilization, which can theoretically extend its shelf-life and further facilitate transportation and storage.

**Figure 4.**
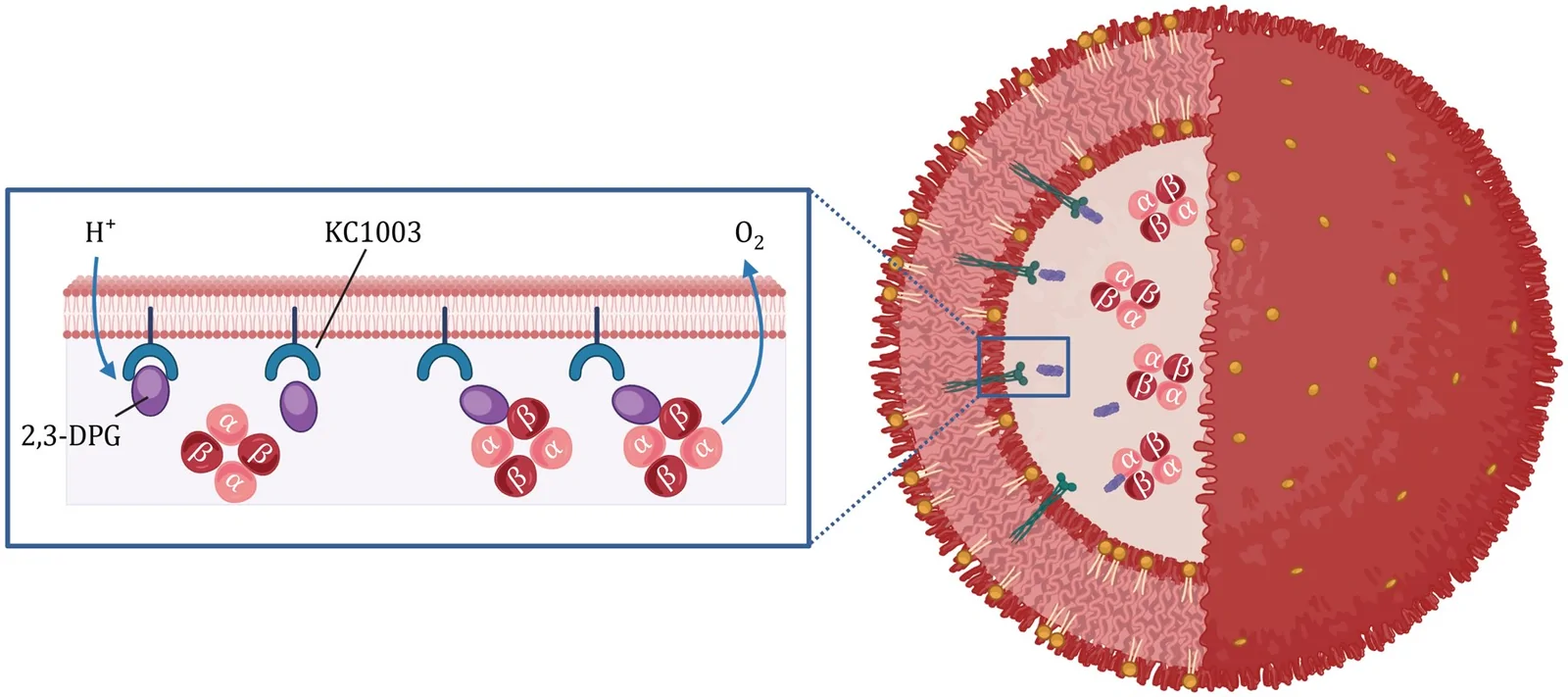
**ErythroMer has a pH-sensitive membrane with KC1003 embedded to modulate the dynamic release of 2,3-DPG, which is engineered to shift its P_50_ in different microenvironments**. Adapted from Zaleski, 2024. Created with Biorender.com.

The pre-clinical studies of ErythroMer are still ongoing (Pan et al., 2016). As one of the most state-of-the-art HBOCs, EM has been placed with high hopes, both by the academics and the US government. This meticulously crafted HBOC is scheduled for assessment in human bodies before the DARPA grant ends in 4 years (DBT Office, 2021; Zaleski, 2024).

### HEMO_2_life

To date, marine organisms have been poorly studied, among which one that lives in the intertidal region exerts special attraction, as the intertidal area symbolizes the transition from sea to land and may answer the questions for the evolution of ways of oxygenation. Franck Zal, who later co-founded the company Hemarina, first found a universal oxygen carrier in *Arenicola marina*, a marine lugworm, by investigating how these organisms breathe in both marine and land environments at high and low tide (Zal et al., 2022). It turns out that the well-evolved Hb, M101, possessed by the worm is naturally polymerized, with its weight up to 3.6 kDa, 56-fold larger than the human one. In order to adapt to the changes between hypoxic and oxygen-rich environment, M101 allows the adaptive on-load and off-load activities modified by Mg^2+^ and Ca^2+^ to ensure a stable oxygen supply to tissues (Batool et al., 2020; Chen et al., 2023; Varney et al., 2021; Zal et al., 2022). It also possesses antioxidative properties due to its intrinsic Cu/Zn-superoxide dismutase (SOD) activity, which may be utilized as an anti-oxygenation additive since it can actively scavenge oxygen radicals (Varney et al., 2021; Zal et al., 2022).

The bioinspired product, HEMO_2_life from Hemarina, is a natural extracellular biopolymer hemoglobin developed from M101. Due to its low P_50_ of 7 mmHg, HEMO_2_life can only release oxygen to hypoxic tissues through passive release under an oxygen gradient. The stability to provide an oxygen-rich environment along with all the attributes as described above has led HEMO_2_life to the development of an additive to organ preservation solutions, which sustains the normoxic condition and the energetic metabolism of the preserved organ.

Several pivotal trials were carried out to evaluate its safety profile as an additive to kidney preservation fluid as well as its efficacy, entailing improvement on patient survival, graft survival, and renal function. The initial OXYOP trial involving 58 paired kidneys successfully met its primary safety objective, demonstrating no occurrence of product-related serious adverse events (SAEs) and comparable adverse event (AE) rates. In accordance with a more rigorous DGF definition (a minimum of two dialysis sessions), the HEMO_2_life arm exhibited a 7% rate in contrast to 26% in the contralateral control group (Le Meur et al., 2020). These data prompted OXYOP2, a phase III study powered for more patients, whose primary objective is to confirm a 15% absolute reduction in DGF (Le Meur et al., 2023). The efficacy and safety of HEMO_2_life are supported by data from OXYOP, and a 4-year patient survival advantage of 98.3% compared to 86% has been demonstrated. HEMO_2_life has received CE marking as a Class III medical device in the European Union for use in organ preservation solutions (Hemarina, 2022). As the first HBOC to obtain such regulatory authorization, it represents a promising technology for wider clinical adoption in organ transplantation.

Not only in the United States, France, and Japan, but we have also witnessed remarkable progress by Chinese teams recently in developing the next generation of HBOCs. YQ23, a cross-linked bovine-derived HBOC from the Bios Biotechnology Co., Ltd., has reached phase I clinical stage based on solid pre-clinical data, which confirmed the protective effects of YQ23 as the resuscitation fluid of uncontrolled hemorrhagic shock (UHS) and its effort in inhibiting tumor growth (Chow et al., 2021; Kuang et al., 2019; 2021; Lee et al., 2015; Li et al., 2017; Qi et al., 2017; Zhang et al., 2021). The company also introduced Biopure’s technology from the United States and holds the full intellectual property rights. XY101 from Xi’an Blood Oxygen Biotechnology Co., Ltd. is entering the clinical stage after recent patent declassification, while PolyCHb from the Chinese Institute of Blood Transfusion is undergoing further investigation (Kong et al., 2023; Zhou et al., 2023). This indicates that the latest HBOC technology has been picking up momentum and gaining more traction worldwide.

## Future implications for use: where are we heading?

With the third-generation HBOCs heading to phase III investigation with great promise, ideal endpoints for on-site hemorrhage interventions still remain controversial for the researchers and the FDA today. Past failures have highlighted the importance of a clear, specific, and pragmatic application scenario before the pivotal trial, without which, the mismatch could fatally impact the data generation, outcome interpretation, and future product positioning. Therefore, it is of paramount importance to specify the exact implications for use to bridge the gap between clinical endpoints of HBOC and its real-world use for future clinical investigation and commercialization.

Here, we summarize common and rational use scenarios pursued by the third-generation products on the bedside, such as oxygen bridge, organ preservation, and veterinary use. We also expect further expansion to emerging fields such as fetal oxygenation and tissue engineering, which require a better solution of gas delivery other than direct infusion or perfluorocarbons (Fig. 5).

**Figure 5.**
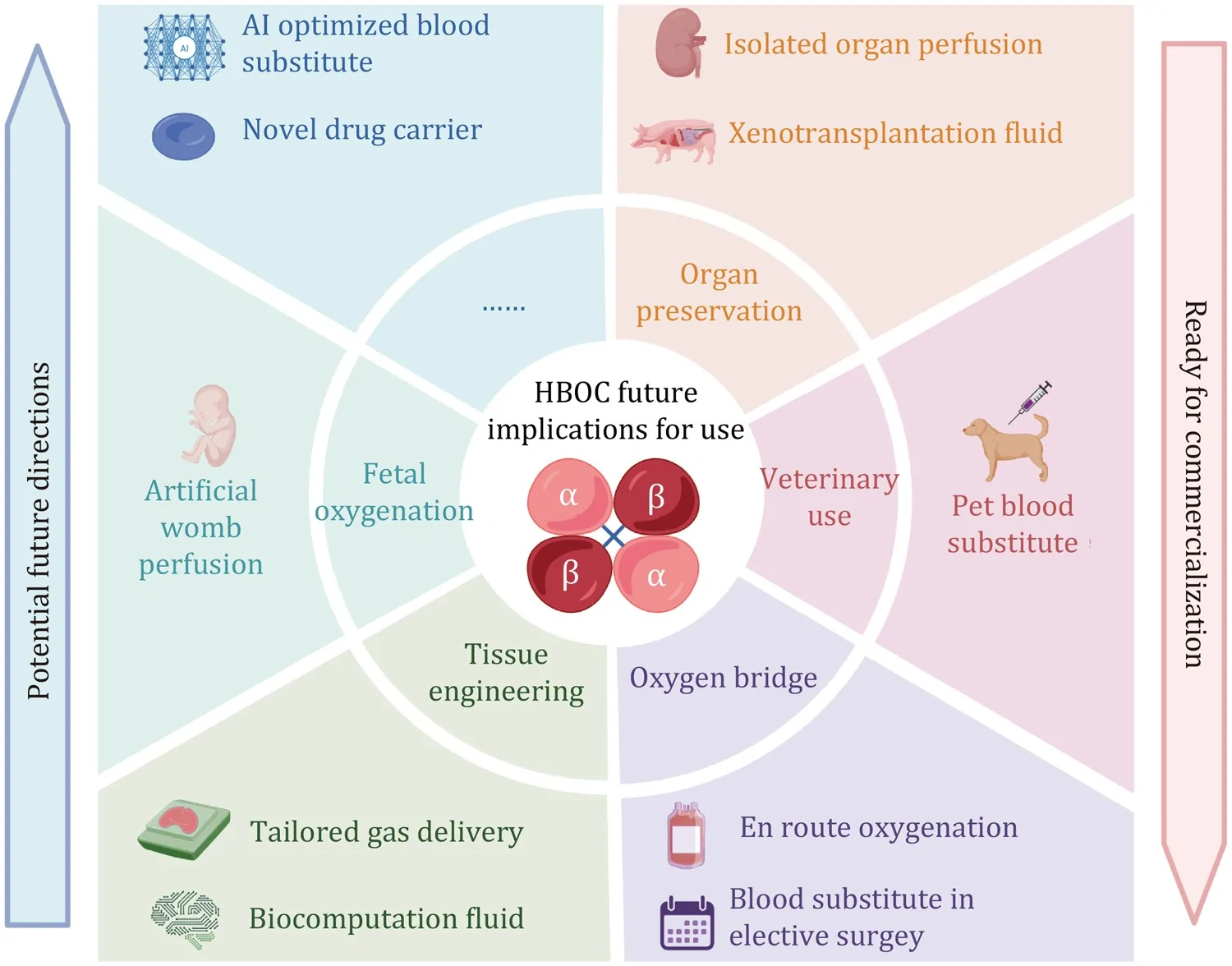
**Future implications for the use of HBOC are summarized with their exact roles to play in clinical or commercial settings**. HBOCs for organ preservation and veterinary use have already entered the market, with oxygen bridge under stricter investigation in clinical trials currently on-going. Future potential roles of HBOC are listed on the left. Created with Biorender.com.

### Oxygen bridge

Since no HBOC has been commercialized in the market, current practices for trauma or hemorrhaging patients to maintain the blood volume are still limited to crystalloids and colloids such as hydroxyethyl starches (HES) and dextran when whole blood or plasma is not available (Butler et al., 2014; Hoffman et al., 2022; Voller et al., 2021). However, the blood volume is sustained without any additional oxygen that is urgently in need, and the application has been found with a higher mortality rate, potential risk of renal failure, longer coagulation time, and several bleeding complications (Butler et al., 2014). According to the transfusion guidelines, RBCs, as the essential oxygen carrier, should be applied under the 10/30 rules (i.e., RBC transfusion is indicated in cases where the Hb concentration is below 10 g/dL or the hematocrit is below 30%) to maximize clinical outcomes and avoid potential transfusion-related risks aforementioned (Adams and Lundy, 1942; García-Roa et al., 2017; Madjdpour et al., 2006). By mimicking the functionality of the natural whole blood, HBOC combined with crystalloids may be sufficient to sustain blood volume while maintaining the Hb and oxygen level. Of note, HBOCs with high P_50_ that are prone to release large amounts of oxygen may not be suitable for initial perfusion as it may cause serious oxidative stress aforementioned.

Further, an indication of oxygen bridge also allows HBOCs to be used in elective surgeries when additional RBCs are not an option during the surgery. Normally, emergency surgeries are given more priority over limited blood resources, while surgeries that can be elective may be excluded from the common blood supply when blood is in short supply. In most countries, patients taking elective surgeries are required to accept autotransfusion or obtain donated blood from family members or other qualified allogeneic donors before the required date (Dupuis et al., 1999; Hameed et al., 2022; Owings et al., 1989; Xue et al., 2016). Unfortunately, in most cases, no additional blood is prepared for use by hospitals if an extra amount of blood is needed in the elective settings, which can be fatal when an unexpected hemorrhage takes place. In this case, HBOC can be their last hope since no fresh RBC can be applied in the middle of the surgery when the prepared amount is used up.

### Organ preservation

After the ability of HBOC to deliver oxygen to hypoxic tissues *in vivo* has been established, *ex vivo* oxygenation of tissues has been logically thought of, leading to its use in organ preservation. Take the kidney, one of the widest transplanted organs, as an example. Kidney transplantation has become the last choice for late-stage renal disease (Owings et al., 1989). However, a series of unmet medical needs still impede a perfect transplantation due to current preservation methods. Common complications observed can be concluded as DGF, which is the manifestation of acute kidney injury (AKI) in transplantation, causing post-transplantation oliguria, a higher rate of graft immunogenicity, and the occurrence of acute rejection (Batool et al., 2020; Fontes et al., 2022; Le Meur et al., 2020; 2023; Lupon et al., 2021; Varney et al., 2021; Zal et al., 2022). Current organ preservation techniques include static cold storage (SCS) and hypothermic machine perfusion (HMP), with HMP exhibiting a lower DGF rate than SCS (Owings et al., 1989; Tatum et al., 2021).

As organs are disconnected from the donors, oxygen and nutrient supplies are abruptly cut off, causing acute anoxia in cells if they function normally (Zal et al., 2022). SCS suppresses the metabolism of cells through low temperature of 4°C, 90% lower than the normal level. However, hypoxia caused by the remaining 10% of metabolism may induce ischemia-reperfusion injury (IRI) induced by large amount of oxygen radicals and cytokine (Fontes et al., 2022; Owings et al., 1989). While HMP, likewise, cannot provide oxygen for the organs with the current preservation fluid (Fontes et al., 2022).

With less immunological concern and a high oxygen affinity, HEMO_2_life has first been approved to be added in UW (University of Wisconsin solution) and HTK (Histidine-Tryptophan-Ketoglutarate solution) solutions to provide an oxygen-rich environment. HEMO_2_life-added perfusion, one of the few approaches to achieve hypothermic oxygenated perfusion (HOPE), has been proved to increase ATP synthesis and induce less expression of oxidative tissue damage markers than the current standard of practice (Tatum et al., 2021).

Looking ahead, as human-derived organ transplantation has long been unable to meet the massive needs, xenotransplantation, having received emerging breakthroughs and attention recently, may be the dominant trend among future allografts. With gene-edited porcine kidney entering the clinical investigation stage with huge support from the FDA, we may envision an exponential growth of demand in organ transplantation fluid (Mallapaty, 2024; Pan et al., 2024; Schmauch et al., 2024; United Therapeutics, 2025). In light of the recent boom, HBOC, with better functionality and less ethical concerns in the xenotransplantation settings, will no doubt become the optimum and the dominant solution to future organ transplantation, and more established products may be extended to the use of organ preservation in the near future.

### Veterinary use

With the mounting interest in pet ownership and the rising emphasis on pet health, the practice of surgery on pets has proportionately increased, with a large amount of veterinary blood in huge demand. Although some developed countries own their blood banks specially for pets, such as Pet Blood Bank UK in Britain (Walton, 2014), most countries do not have a steady supply of blood for veterinary use. Current solutions lie in donation appeal on social media and black-market deals. However, whether the pet blood bank is locally established or not, it remains challenging for animal blood donation to identify suitable donors, and the recruitment process is often costly, especially in canine cases, as dogs have as many as eight common blood types. Furthermore, studies have demonstrated that the majority of pet owners are unaware of the existence of pet blood banks and the possibility of pet blood donation. This lack of awareness makes it even harder to recruit donors in emergency situations (Wilder and Humm, 2019).

With the advent of HBOC, a portable and universal solution for clinical use in veterinary surgery demonstrates huge potential. Based on the existing robust pre-clinical profile on numerous kinds of animals, with rats, dogs, and cats included, current HBOCs under ongoing evaluation can be easily expanded to veterinary use without further validation. HBOC-301 (Oxyglobin) from Hemopure has already been approved for pets, delivering new hope for tens of thousands of desperate pet owners.

### Fetal oxygenation

Current SOP of oxygenation to premature neonates with respiratory failure depends on the conventional extracorporeal membrane oxygenation (ECMO), which imposes huge ventilation pressure on the immature neonatal lungs, along with a higher incidence rate of intracranial hemorrhage and many other irreversible damages (Partridge et al., 2017; FDA, 2023). Although novel treatments such as artificial womb and artificial placenta attempt to mitigate the direct pressure through cordlike transfusion, the direct infusion of blended gas through oxygenator still renders several defects such as progressive fall in Hb and following anemia (McLeod et al., 2019; Partridge et al., 2017; FDA, 2023). Since the natural fetal Hb (HbF) possesses higher affinity for oxygen and delivers gas through the gradient of P_50_ between the mother and the embryo, HBOCs with altered P_50_ around 22 mmHg can be applied to mimic the natural oxygenation through blood circulation, with theoretically milder ventilation pressure and less operational complexity. As PFCs have already been proposed to be applied in the artificial placenta, it is believed that HBOC with longer stability time and better functionality should outperform (Spencer and Mychaliska, 2022).

### Tissue engineering

Ex-situ tissue engineering and the potential deployment of large-scale organoids in the near future will also require massive amounts of stable fluids with low cost for oxygenation, rather than the limited human-derived Hb with ethical concerns of application on non-human organisms (FDA, 2025). From tissue regeneration to organoids for drug testing, emerging tissue engineering techniques are calling for novel oxygen delivery systems. Without a physiological-like vascular system to guide the oxygen distribution, direct augmented gas perfusion may not satisfy the varying oxygen demand from different types of cells, leading to regional hyperoxia and hypoxia (Zoneff et al., 2024). The sudden surge of oxygen level also triggers the inadvertent ROS overproduction (Mendez-Romero et al., 2022). The extraction of oxygen should be conducted through the natural gradient elicited by the tissue demand instead of imposing oxygen tension. In light of the ability to reversibly bind oxygen, the application of HBOC can well emulate the proper physiological environment to provide tailored oxygen delivery. Moreover, HBOC can be further engineered as a multi-potent gas carrier to meet the metabolic needs of removing wasted metabolites, which may further simplify the construction of *ex-situ* metabolic networks. For instance, wetware computing and organoid intelligence, with recent progress, may benefit from the application of HBOCs. As a promising alternative to the energy-consuming hardware computing, biocomputation requires meticulously designed microfluidics for nutrient delivery, in the absence of which, the passive diffusion may not satisfy the optimal oxygen demand and thus accelerates the central cell death (Jordan et al., 2024; Kagan et al., 2022). With the combination of the “organ-on-a-chip” systems, the artificial vasculature network can closely match the cellular oxygen uptake and achieve the tailored nutrient delivery.

### Al-optimized blood substitute

With RFdiffusion being able to design novel proteins, it is likely that we could envisage the fourth generation HBOC (Watson et al., 2023; Lauko et al., 2025). Based on the understanding of the 3,600 kDa, integrated and highly internally rigid natural protein M101 from HEMO_2_life, new recombinant proteins can be redesigned or created using the emerging artificial intelligence (AI). Traditional chemical methods can be replaced with *de novo* design of integrated multi-unit proteins with more oxygen binding sites, combined with increasingly sophisticated genetic and protein engineering for synthetic biology to produce recombinant Hb on a larger scale, instead of extracting it from natural wasted blood. At the same time, *de novo* design of biologically viable enzymes has also shown great promise (Watson et al., 2023). Active sites can be integrated into pre-designed protein structures, with enzymes such as 2,3-DPG, SOD, etc., being re-engineered and incorporated into the binding pockets of macromolecular proteins. Combined with the known pH-sensing units and mechanisms, the dynamic function of natural RBCs can be further imitated. All these approaches will not only further reduce the immunogenicity of the traditionally applied polymers and solve the issue of hemoglobin disintegration, but also hold the promise of surpassing the performance of natural RBCs with higher oxygen transport efficiency, thereby reducing the problem of natural defects such as iron overload caused by excess transfusion.

HBOC is much more than the potency itself. It has a huge role to play in the prognosis of trauma and the management of blood resources, as well as in the regulation of the blood market and the elimination of disparity in medical resources between different socio-economic classes. The repeated failures of previous generations of products highlight the need for ethical considerations in trial design, well-informed approval processes, and comprehensive guidance on use scenarios to receive not only financial support but also endorsement and guidance from the government and regulatory authorities. We have already surpassed the most challenging stage of exploration from the beginning, a period when we managed to envision and conceive the promising future, but also wasted several valuable products, such as PolyHeme due to our lack of recognition and preparation. After seeking out the right position, HBOC should receive wider attention and support from all medical communities and continue to be tested in the clinical use of various departments. Relevant guidelines should be jointly drafted to regulate its application and more detailed use scenarios as well. After decades of pursuit of a portable, universal, easy-to-preserve oxygen carrier, a viable product finally appears to be on the horizon. From understanding the underlying mechanism through failures to accepting the side-effect profile, it is by no means easy to develop a desirable HBOC and realize its proper indication for use. Despite several apprehensions and ethical concerns from the public and regulatory authorities, HBOC is sure to bring glimpses of hope to whom in desperate need and imponderable benefit to the whole society.

## Artificial intelligence

The authors declare that this work was completed without the use of AI-assisted technologies.

## Abbreviations

2,3-DPG: 2,3-diphosphoglycerate
AE: adverse effect
AKI: acute kidney injury
BEL-A: Bristol Erythroid Line Adult
BLA: Biologic License Application
CAT: catalase
CB: cord blood
COP: colloid osmotic pressure
DAMP: damage-associated molecular pattern
DARPA: Defense Advanced Research Projects Agency
DGF: delayed draft function
ECMO: extracorporeal membrane oxygenation
EU: European Union
FDA: Food and Drug Administration
FSHARP: Fieldable Solutions for Hemorrhage with bio-Artificial Resuscitation Products
HBOC: hemoglobin-based oxygen carrier
HES: hydroxyethyl starches
HMP: hypothermic machine perfusion
HOPE: hypothermic oxygenated perfusion
HTK: Histidine-Tryptophan-Ketoglutarate solution
Hb: hemoglobin
HbF: fetal hemoglobin
HbV: hemoglobin vesicles
IRI: ischemia-reperfusion injury
iPSC: induced pluripotent stem cells
LMB: leucomethylene blue
LMIC: low- and middle-income countries
MOF: multiple organ failure
p50: oxygen affinity when Hb is half saturated
PB: peripheral blood
PEG: polyethylene glycol
PFC: perfluorocarbon
PLP: pyridoxal 5′-phosphate
PRBC: packed red blood cell
RBC: red blood cell
SAE: serious adverse effect
SCS: static cold storage
SOD: superoxide dismutase
UW: University of Wisconsin solution
